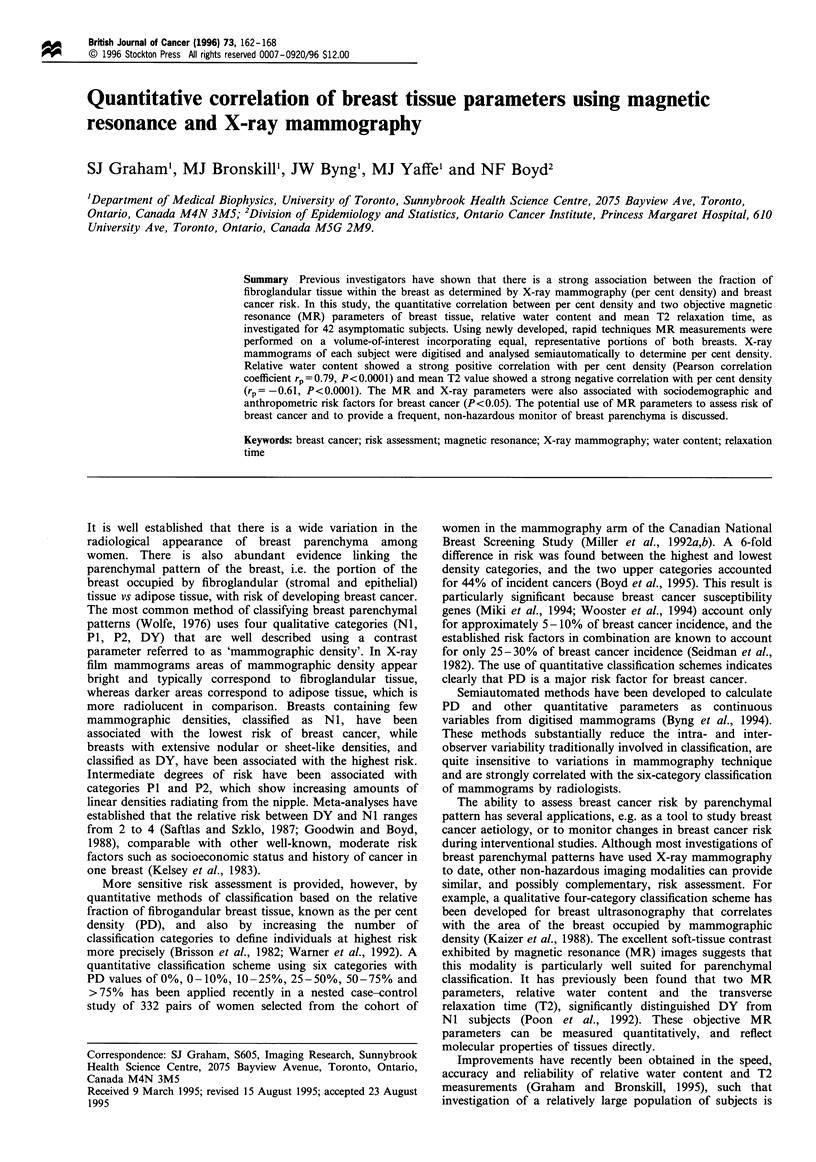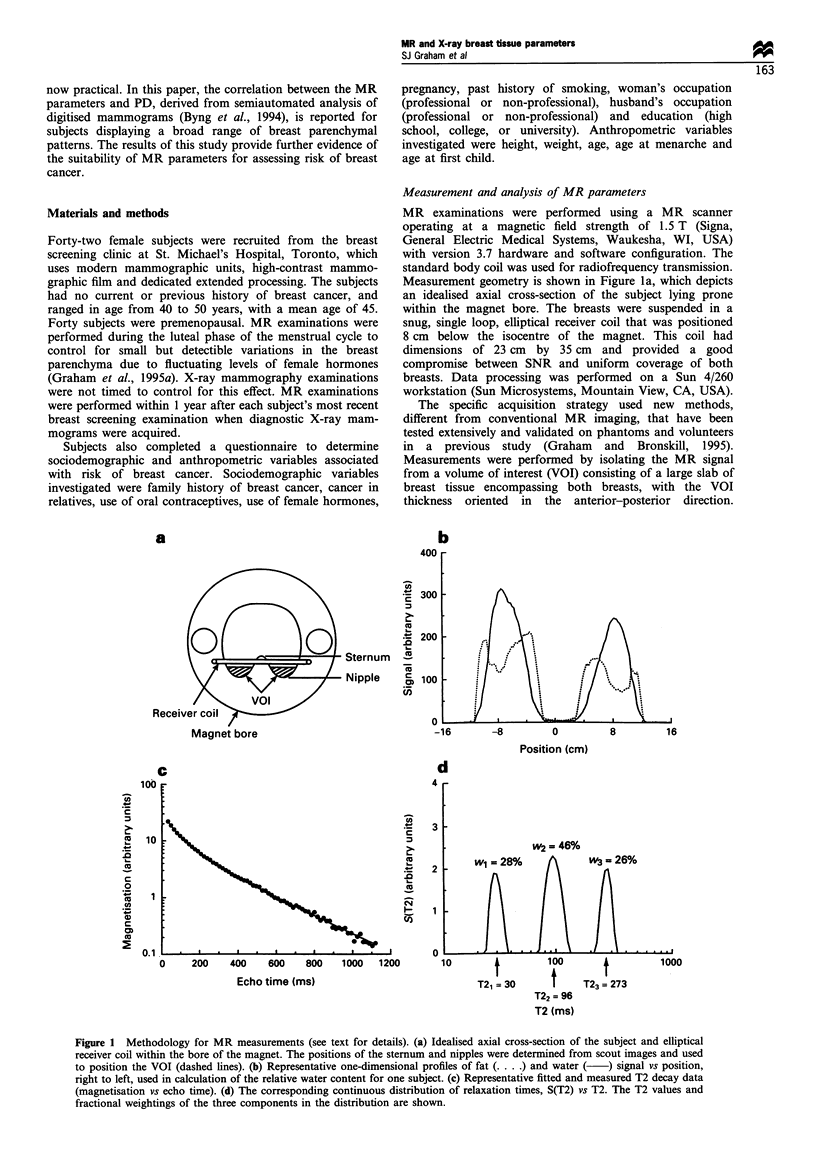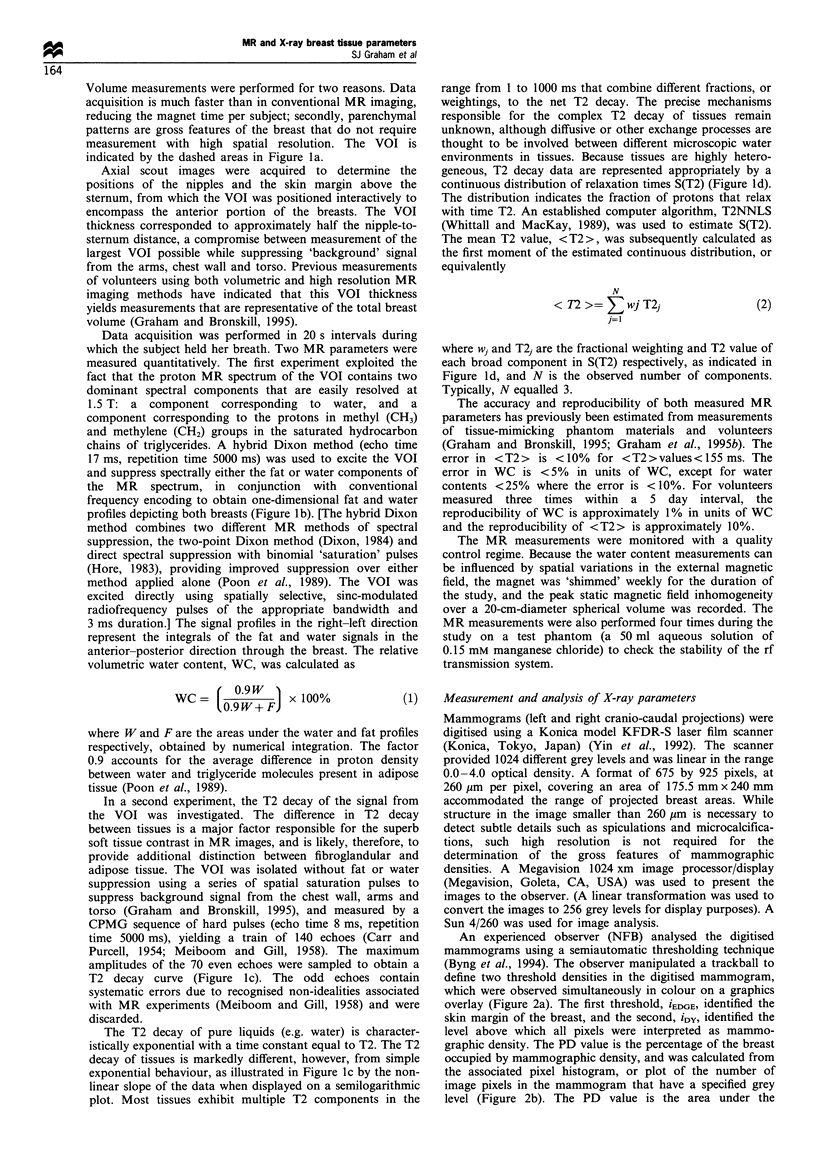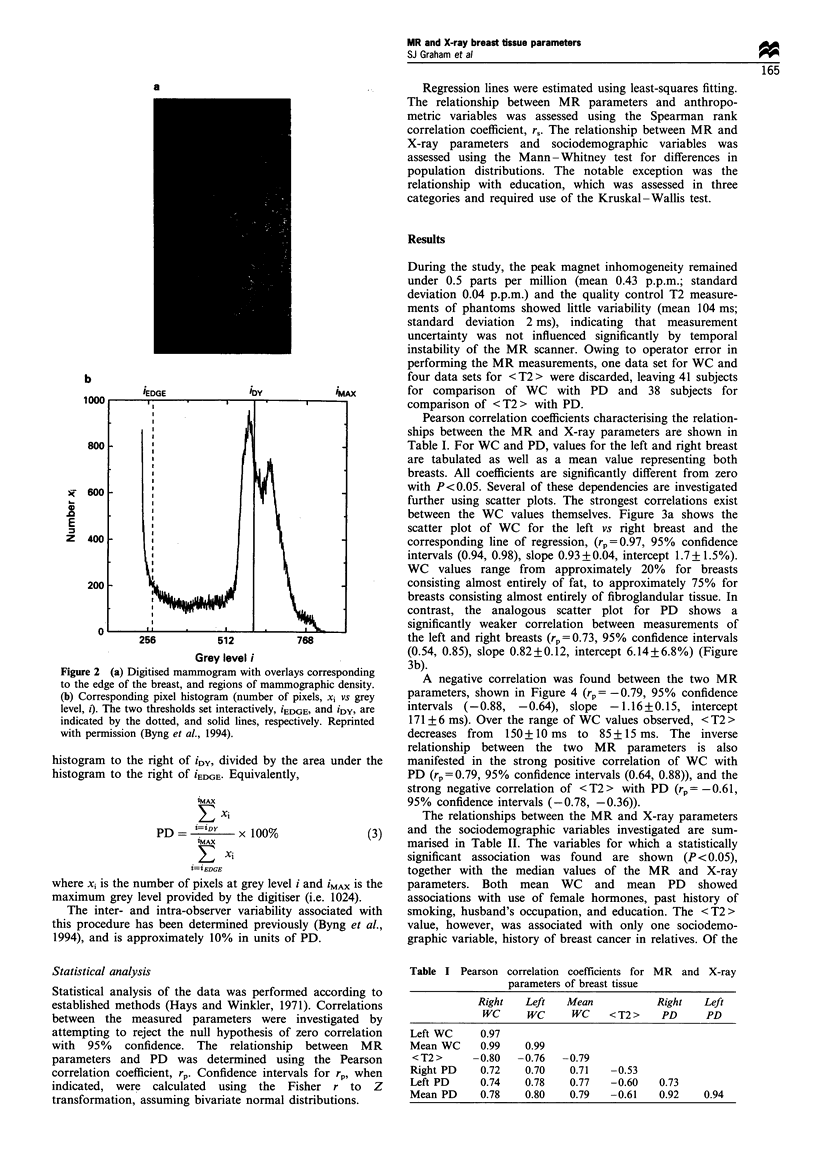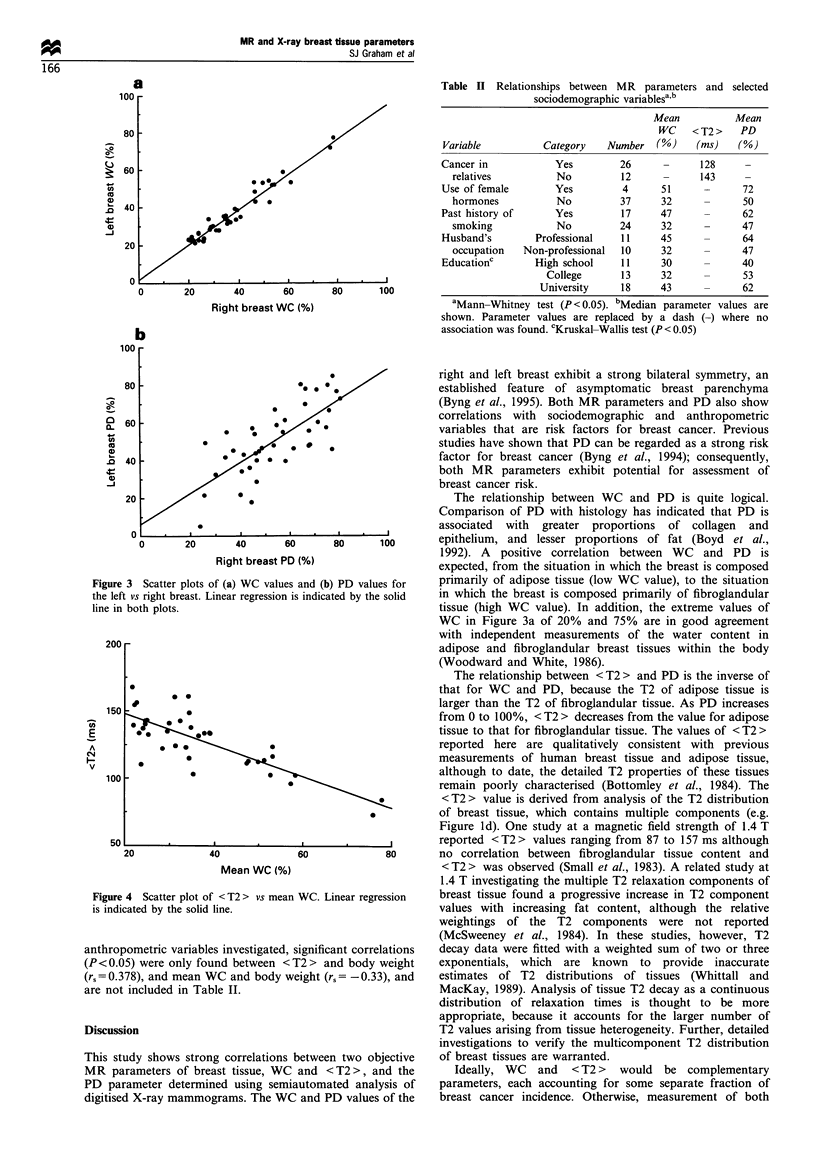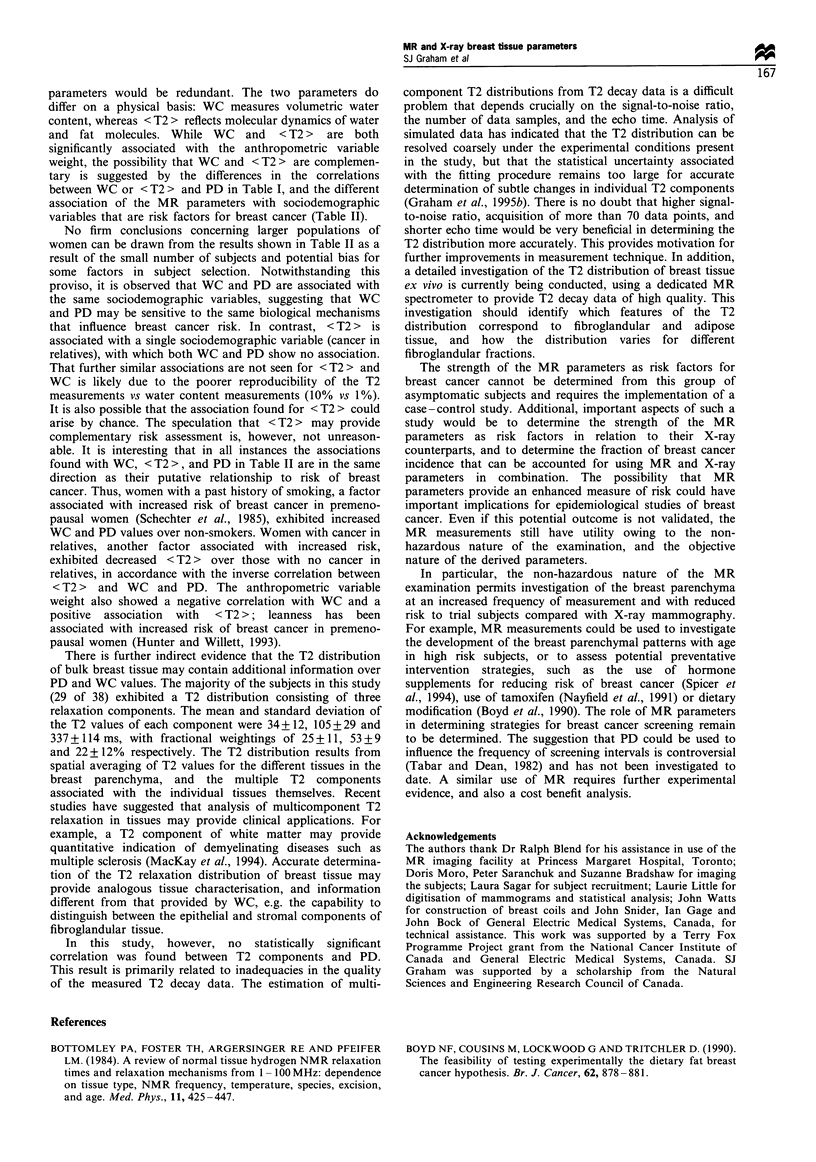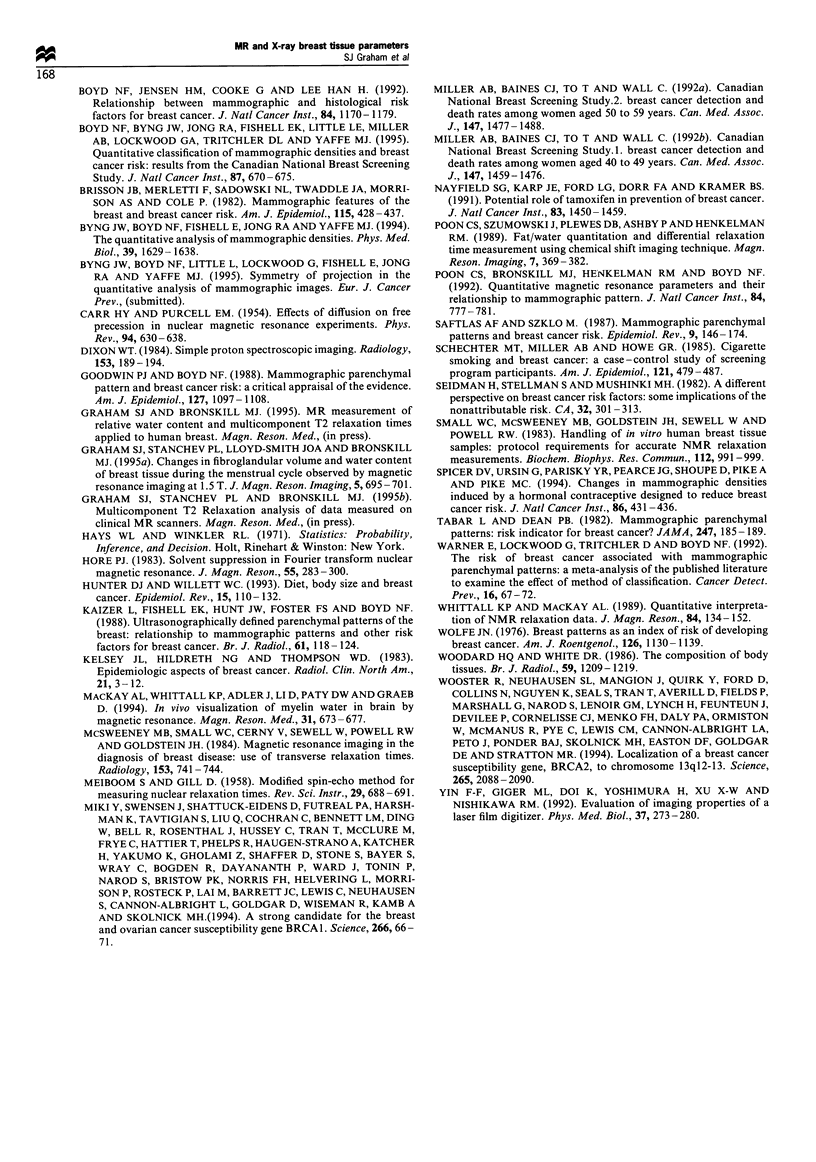# Quantitative correlation of breast tissue parameters using magnetic resonance and X-ray mammography.

**DOI:** 10.1038/bjc.1996.30

**Published:** 1996-01

**Authors:** S. J. Graham, M. J. Bronskill, J. W. Byng, M. J. Yaffe, N. F. Boyd

**Affiliations:** Department of Medical Biophysics, University of Toronto, Sunnybrook Health Science Centre, Ontario, Canada.

## Abstract

**Images:**


British Journal of Cancer (1996) 73, 162-168
© 1996 Stockton Press All rights reserved 0007-0920/96 $12.00

### Quantitative correlation of breast tissue parameters using magnetic resonance and X-ray mammography

SJ Graham[1], MJ Bronskill[1], JW Byng[1], MJ Yaffe[1] and NF Boyd[2]

[1]*Department of Medical Biophysics, University of Toronto, Sunnybrook Health Science Centre, 2075 Bayview Ave, Toronto, Ontario, Canada M4N 3M5;* [2]*Division of Epidemiology and Statistics, Ontario Cancer Institute, Princess Margaret Hospital, 610 University Ave, Toronto, Ontario, Canada M5G 2M9.*

**Summary** Previous investigators have shown that there is a strong association between the fraction of fibroglandular tissue within the breast as determined by X-ray mammography (per cent density) and breast cancer risk. In this study, the quantitative correlation between per cent density and two objective magnetic resonance (MR) parameters of breast tissue, relative water content and mean T2 relaxation time, as investigated for 42 asymptomatic subjects. Using newly developed, rapid techniques MR measurements were performed on a volume-of-interest incorporating equal, representative portions of both breasts. X-ray mammograms of each subject were digitised and analysed semiautomatically to determine per cent density. Relative water content showed a strong positive correlation with per cent density (Pearson correlation coefficient $r_p = 0.79$, $P < 0.0001$) and mean T2 value showed a strong negative correlation with per cent density ($r_p = -0.61$, $P < 0.0001$). The MR and X-ray parameters were also associated with sociodemographic and anthropometric risk factors for breast cancer ($P < 0.05$). The potential use of MR parameters to assess risk of breast cancer and to provide a frequent, non-hazardous monitor of breast parenchyma is discussed.

**Keywords:** breast cancer; risk assessment; magnetic resonance; X-ray mammography; water content; relaxation time

It is well established that there is a wide variation in the radiological appearance of breast parenchyma among women. There is also abundant evidence linking the parenchymal pattern of the breast, i.e. the portion of the breast occupied by fibroglandular (stromal and epithelial) tissue *vs* adipose tissue, with risk of developing breast cancer. The most common method of classifying breast parenchymal patterns (Wolfe, 1976) uses four qualitative categories (N1, P1, P2, DY) that are well described using a contrast parameter referred to as 'mammographic density'. In X-ray film mammograms areas of mammographic density appear bright and typically correspond to fibroglandular tissue, whereas darker areas correspond to adipose tissue, which is more radiolucent in comparison. Breasts containing few mammographic densities, classified as N1, have been associated with the lowest risk of breast cancer, while breasts with extensive nodular or sheet-like densities, and classified as DY, have been associated with the highest risk. Intermediate degrees of risk have been associated with categories P1 and P2, which show increasing amounts of linear densities radiating from the nipple. Meta-analyses have established that the relative risk between DY and N1 ranges from 2 to 4 (Saftlas and Szklo, 1987; Goodwin and Boyd, 1988), comparable with other well-known, moderate risk factors such as socioeconomic status and history of cancer in one breast (Kelsey *et al.*, 1983).

More sensitive risk assessment is provided, however, by quantitative methods of classification based on the relative fraction of fibrogandular breast tissue, known as the per cent density (PD), and also by increasing the number of classification categories to define individuals at highest risk more precisely (Brisson *et al.*, 1982; Warner *et al.*, 1992). A quantitative classification scheme using six categories with PD values of 0%, 0-10%, 10-25%, 25-50%, 50-75% and >75% has been applied recently in a nested case–control study of 332 pairs of women selected from the cohort of women in the mammography arm of the Canadian National Breast Screening Study (Miller *et al.*, 1992*a,b*). A 6-fold difference in risk was found between the highest and lowest density categories, and the two upper categories accounted for 44% of incident cancers (Boyd *et al.*, 1995). This result is particularly significant because breast cancer susceptibility genes (Miki *et al.*, 1994; Wooster *et al.*, 1994) account only for approximately 5-10% of breast cancer incidence, and the established risk factors in combination are known to account for only 25-30% of breast cancer incidence (Seidman *et al.*, 1982). The use of quantitative classification schemes indicates clearly that PD is a major risk factor for breast cancer.

Semiautomated methods have been developed to calculate PD and other quantitative parameters as continuous variables from digitised mammograms (Byng *et al.*, 1994). These methods substantially reduce the intra- and inter-observer variability traditionally involved in classification, are quite insensitive to variations in mammography technique and are strongly correlated with the six-category classification of mammograms by radiologists.

The ability to assess breast cancer risk by parenchymal pattern has several applications, e.g. as a tool to study breast cancer aetiology, or to monitor changes in breast cancer risk during interventional studies. Although most investigations of breast parenchymal patterns have used X-ray mammography to date, other non-hazardous imaging modalities can provide similar, and possibly complementary, risk assessment. For example, a qualitative four-category classification scheme has been developed for breast ultrasonography that correlates with the area of the breast occupied by mammographic density (Kaizer *et al.*, 1988). The excellent soft-tissue contrast exhibited by magnetic resonance (MR) images suggests that this modality is particularly well suited for parenchymal classification. It has previously been found that two MR parameters, relative water content and the transverse relaxation time (T2), significantly distinguished DY from N1 subjects (Poon *et al.*, 1992). These objective MR parameters can be measured quantitatively, and reflect molecular properties of tissues directly.

Improvements have recently been obtained in the speed, accuracy and reliability of relative water content and T2 measurements (Graham and Bronskill, 1995), such that investigation of a relatively large population of subjects is

Correspondence: SJ Graham, S605, Imaging Research, Sunnybrook Health Science Centre, 2075 Bayview Avenue, Toronto, Ontario, Canada M4N 3M5
Received 9 March 1995; revised 15 August 1995; accepted 23 August 1995

now practical. In this paper, the correlation between the MR parameters and PD, derived from semiautomated analysis of digitised mammograms (Byng *et al.*, 1994), is reported for subjects displaying a broad range of breast parenchymal patterns. The results of this study provide further evidence of the suitability of MR parameters for assessing risk of breast cancer.

### Materials and methods

Forty-two female subjects were recruited from the breast screening clinic at St. Michael's Hospital, Toronto, which uses modern mammographic units, high-contrast mammographic film and dedicated extended processing. The subjects had no current or previous history of breast cancer, and ranged in age from 40 to 50 years, with a mean age of 45. Forty subjects were premenopausal. MR examinations were performed during the luteal phase of the menstrual cycle to control for small but detectible variations in the breast parenchyma due to fluctuating levels of female hormones (Graham *et al.*, 1995*a*). X-ray mammography examinations were not timed to control for this effect. MR examinations were performed within 1 year after each subject's most recent breast screening examination when diagnostic X-ray mammograms were acquired.

Subjects also completed a questionnaire to determine sociodemographic and anthropometric variables associated with risk of breast cancer. Sociodemographic variables investigated were family history of breast cancer, cancer in relatives, use of oral contraceptives, use of female hormones, pregnancy, past history of smoking, woman's occupation (professional or non-professional), husband's occupation (professional or non-professional) and education (high school, college, or university). Anthropometric variables investigated were height, weight, age, age at menarche and age at first child.

### *Measurement and analysis of MR parameters*

MR examinations were performed using a MR scanner operating at a magnetic field strength of 1.5 T (Signa, General Electric Medical Systems, Waukesha, WI, USA) with version 3.7 hardware and software configuration. The standard body coil was used for radiofrequency transmission. Measurement geometry is shown in Figure 1a, which depicts an idealised axial cross-section of the subject lying prone within the magnet bore. The breasts were suspended in a snug, single loop, elliptical receiver coil that was positioned 8 cm below the isocentre of the magnet. This coil had dimensions of 23 cm by 35 cm and provided a good compromise between SNR and uniform coverage of both breasts. Data processing was performed on a Sun 4/260 workstation (Sun Microsystems, Mountain View, CA, USA).

The specific acquisition strategy used new methods, different from conventional MR imaging, that have been tested extensively and validated on phantoms and volunteers in a previous study (Graham and Bronskill, 1995). Measurements were performed by isolating the MR signal from a volume of interest (VOI) consisting of a large slab of breast tissue encompassing both breasts, with the VOI thickness oriented in the anterior–posterior direction.

**Figure 1** Methodology for MR measurements (see text for details). (**a**) Idealised axial cross-section of the subject and elliptical receiver coil within the bore of the magnet. The positions of the sternum and nipples were determined from scout images and used to position the VOI (dashed lines). (**b**) Representative one-dimensional profiles of fat (. . . .) and water (——) signal *vs* position, right to left, used in calculation of the relative water content for one subject. (**c**) Representative fitted and measured T2 decay data (magnetisation *vs* echo time). (**d**) The corresponding continuous distribution of relaxation times, S(T2) *vs* T2. The T2 values and fractional weightings of the three components in the distribution are shown.

Volume measurements were performed for two reasons. Data acquisition is much faster than in conventional MR imaging, reducing the magnet time per subject; secondly, parenchymal patterns are gross features of the breast that do not require measurement with high spatial resolution. The VOI is indicated by the dashed areas in Figure 1a.

Axial scout images were acquired to determine the positions of the nipples and the skin margin above the sternum, from which the VOI was positioned interactively to encompass the anterior portion of the breasts. The VOI thickness corresponded to approximately half the nipple-to-sternum distance, a compromise between measurement of the largest VOI possible while suppressing 'background' signal from the arms, chest wall and torso. Previous measurements of volunteers using both volumetric and high resolution MR imaging methods have indicated that this VOI thickness yields measurements that are representative of the total breast volume (Graham and Bronskill, 1995).

Data acquisition was performed in 20 s intervals during which the subject held her breath. Two MR parameters were measured quantitatively. The first experiment exploited the fact that the proton MR spectrum of the VOI contains two dominant spectral components that are easily resolved at 1.5 T: a component corresponding to water, and a component corresponding to the protons in methyl ($CH_3$) and methylene ($CH_2$) groups in the saturated hydrocarbon chains of triglycerides. A hybrid Dixon method (echo time 17 ms, repetition time 5000 ms) was used to excite the VOI and suppress spectrally either the fat or water components of the MR spectrum, in conjunction with conventional frequency encoding to obtain one-dimensional fat and water profiles depicting both breasts (Figure 1b). [The hybrid Dixon method combines two different MR methods of spectral suppression, the two-point Dixon method (Dixon, 1984) and direct spectral suppression with binomial 'saturation' pulses (Hore, 1983), providing improved suppression over either method applied alone (Poon et al., 1989). The VOI was excited directly using spatially selective, sinc-modulated radiofrequency pulses of the appropriate bandwidth and 3 ms duration.] The signal profiles in the right–left direction represent the integrals of the fat and water signals in the anterior–posterior direction through the breast. The relative volumetric water content, WC, was calculated as

$$WC = \left(\frac{0.9W}{0.9W + F}\right) \times 100\% \qquad (1)$$

where $W$ and $F$ are the areas under the water and fat profiles respectively, obtained by numerical integration. The factor 0.9 accounts for the average difference in proton density between water and triglyceride molecules present in adipose tissue (Poon et al., 1989).

In a second experiment, the T2 decay of the signal from the VOI was investigated. The difference in T2 decay between tissues is a major factor responsible for the superb soft tissue contrast in MR images, and is likely, therefore, to provide additional distinction between fibroglandular and adipose tissue. The VOI was isolated without fat or water suppression using a series of spatial saturation pulses to suppress background signal from the chest wall, arms and torso (Graham and Bronskill, 1995), and measured by a CPMG sequence of hard pulses (echo time 8 ms, repetition time 5000 ms), yielding a train of 140 echoes (Carr and Purcell, 1954; Meiboom and Gill, 1958). The maximum amplitudes of the 70 even echoes were sampled to obtain a T2 decay curve (Figure 1c). The odd echoes contain systematic errors due to recognised non-idealities associated with MR experiments (Meiboom and Gill, 1958) and were discarded.

The T2 decay of pure liquids (e.g. water) is characteristically exponential with a time constant equal to T2. The T2 decay of tissues is markedly different, however, from simple exponential behaviour, as illustrated in Figure 1c by the non-linear slope of the data when displayed on a semilogarithmic plot. Most tissues exhibit multiple T2 components in the range from 1 to 1000 ms that combine different fractions, or weightings, to the net T2 decay. The precise mechanisms responsible for the complex T2 decay of tissues remain unknown, although diffusive or other exchange processes are thought to be involved between different microscopic water environments in tissues. Because tissues are highly heterogeneous, T2 decay data are represented appropriately by a continuous distribution of relaxation times S(T2) (Figure 1d). The distribution indicates the fraction of protons that relax with time T2. An established computer algorithm, T2NNLS (Whittall and MacKay, 1989), was used to estimate S(T2). The mean T2 value, $<T2>$, was subsequently calculated as the first moment of the estimated continuous distribution, or equivalently

$$< T2 >= \sum_{j=1}^{N} wj\, T2_j \qquad (2)$$

where $w_j$ and $T2_j$ are the fractional weighting and T2 value of each broad component in S(T2) respectively, as indicated in Figure 1d, and $N$ is the observed number of components. Typically, $N$ equalled 3.

The accuracy and reproducibility of both measured MR parameters has previously been estimated from measurements of tissue-mimicking phantom materials and volunteers (Graham and Bronskill, 1995; Graham et al., 1995b). The error in $<T2>$ is $<10\%$ for $<T2>$values$<155$ ms. The error in WC is $<5\%$ in units of WC, except for water contents $<25\%$ where the error is $<10\%$. For volunteers measured three times within a 5 day interval, the reproducibility of WC is approximately 1% in units of WC and the reproducibility of $<T2>$ is approximately 10%.

The MR measurements were monitored with a quality control regime. Because the water content measurements can be influenced by spatial variations in the external magnetic field, the magnet was 'shimmed' weekly for the duration of the study, and the peak static magnetic field inhomogeneity over a 20-cm-diameter spherical volume was recorded. The MR measurements were also performed four times during the study on a test phantom (a 50 ml aqueous solution of 0.15 mM manganese chloride) to check the stability of the rf transmission system.

*Measurement and analysis of X-ray parameters*

Mammograms (left and right cranio-caudal projections) were digitised using a Konica model KFDR-S laser film scanner (Konica, Tokyo, Japan) (Yin et al., 1992). The scanner provided 1024 different grey levels and was linear in the range 0.0–4.0 optical density. A format of 675 by 925 pixels, at 260 $\mu$m per pixel, covering an area of 175.5 mm × 240 mm accommodated the range of projected breast areas. While structure in the image smaller than 260 $\mu$m is necessary to detect subtle details such as spiculations and microcalcifications, such high resolution is not required for the determination of the gross features of mammographic densities. A Megavision 1024 xm image processor/display (Megavision, Goleta, CA, USA) was used to present the images to the observer. (A linear transformation was used to convert the images to 256 grey levels for display purposes). A Sun 4/260 was used for image analysis.

An experienced observer (NFB) analysed the digitised mammograms using a semiautomatic thresholding technique (Byng et al., 1994). The observer manipulated a trackball to define two threshold densities in the digitised mammogram, which were observed simultaneously in colour on a graphics overlay (Figure 2a). The first threshold, $i_{EDGE}$, identified the skin margin of the breast, and the second, $i_{DY}$, identified the level above which all pixels were interpreted as mammographic density. The PD value is the percentage of the breast occupied by mammographic density, and was calculated from the associated pixel histogram, or plot of the number of image pixels in the mammogram that have a specified grey level (Figure 2b). The PD value is the area under the

**Figure 2** (**a**) Digitised mammogram with overlays corresponding to the edge of the breast, and regions of mammographic density. (**b**) Corresponding pixel histogram (number of pixels, $x_i$ *vs* grey level, *i*). The two thresholds set interactively, $i_{EDGE}$, and $i_{DY}$, are indicated by the dotted, and solid lines, respectively. Reprinted with permission (Byng *et al.*, 1994).

histogram to the right of $i_{DY}$, divided by the area under the histogram to the right of $i_{EDGE}$. Equivalently,

$$PD = \frac{\sum_{i=i_{DY}}^{i_{MAX}} x_i}{\sum_{i=i_{EDGE}}^{i_{MAX}} x_i} \times 100\% \quad (3)$$

where $x_i$ is the number of pixels at grey level *i* and $i_{MAX}$ is the maximum grey level provided by the digitiser (i.e. 1024).

The inter- and intra-observer variability associated with this procedure has been determined previously (Byng *et al.*, 1994), and is approximately 10% in units of PD.

### *Statistical analysis*

Statistical analysis of the data was performed according to established methods (Hays and Winkler, 1971). Correlations between the measured parameters were investigated by attempting to reject the null hypothesis of zero correlation with 95% confidence. The relationship between MR parameters and PD was determined using the Pearson correlation coefficient, $r_p$. Confidence intervals for $r_p$, when indicated, were calculated using the Fisher *r* to *Z* transformation, assuming bivariate normal distributions.

Regression lines were estimated using least-squares fitting. The relationship between MR parameters and anthropometric variables was assessed using the Spearman rank correlation coefficient, $r_s$. The relationship between MR and X-ray parameters and sociodemographic variables was assessed using the Mann–Whitney test for differences in population distributions. The notable exception was the relationship with education, which was assessed in three categories and required use of the Kruskal–Wallis test.

### Results

During the study, the peak magnet inhomogeneity remained under 0.5 parts per million (mean 0.43 p.p.m.; standard deviation 0.04 p.p.m.) and the quality control T2 measurements of phantoms showed little variability (mean 104 ms; standard deviation 2 ms), indicating that measurement uncertainty was not influenced significantly by temporal instability of the MR scanner. Owing to operator error in performing the MR measurements, one data set for WC and four data sets for $<T2>$ were discarded, leaving 41 subjects for comparison of WC with PD and 38 subjects for comparison of $<T2>$ with PD.

Pearson correlation coefficients characterising the relationships between the MR and X-ray parameters are shown in Table I. For WC and PD, values for the left and right breast are tabulated as well as a mean value representing both breasts. All coefficients are significantly different from zero with $P<0.05$. Several of these dependencies are investigated further using scatter plots. The strongest correlations exist between the WC values themselves. Figure 3a shows the scatter plot of WC for the left *vs* right breast and the corresponding line of regression, ($r_p=0.97$, 95% confidence intervals (0.94, 0.98), slope $0.93 \pm 0.04$, intercept $1.7 \pm 1.5\%$). WC values range from approximately 20% for breasts consisting almost entirely of fat, to approximately 75% for breasts consisting almost entirely of fibroglandular tissue. In contrast, the analogous scatter plot for PD shows a significantly weaker correlation between measurements of the left and right breasts ($r_p=0.73$, 95% confidence intervals (0.54, 0.85), slope $0.82 \pm 0.12$, intercept $6.14 \pm 6.8\%$) (Figure 3b).

A negative correlation was found between the two MR parameters, shown in Figure 4 ($r_p=-0.79$, 95% confidence intervals ($-0.88$, $-0.64$), slope $-1.16 \pm 0.15$, intercept $171 \pm 6$ ms). Over the range of WC values observed, $<T2>$ decreases from $150 \pm 10$ ms to $85 \pm 15$ ms. The inverse relationship between the two MR parameters is also manifested in the strong positive correlation of WC with PD ($r_p=0.79$, 95% confidence intervals (0.64, 0.88)), and the strong negative correlation of $<T2>$ with PD ($r_p=-0.61$, 95% confidence intervals ($-0.78$, $-0.36$)).

The relationships between the MR and X-ray parameters and the sociodemographic variables investigated are summarised in Table II. The variables for which a statistically significant association was found are shown ($P<0.05$), together with the median values of the MR and X-ray parameters. Both mean WC and mean PD showed associations with use of female hormones, past history of smoking, husband's occupation, and education. The $<T2>$ value, however, was associated with only one sociodemographic variable, history of breast cancer in relatives. Of the

**Table I** Pearson correlation coefficients for MR and X-ray parameters of breast tissue

| | *Right WC* | *Left WC* | *Mean WC* | $<T2>$ | *Right PD* | *Left PD* |
|---|---|---|---|---|---|---|
| Left WC | 0.97 | | | | | |
| Mean WC | 0.99 | 0.99 | | | | |
| $<T2>$ | −0.80 | −0.76 | −0.79 | | | |
| Right PD | 0.72 | 0.70 | 0.71 | −0.53 | | |
| Left PD | 0.74 | 0.78 | 0.77 | −0.60 | 0.73 | |
| Mean PD | 0.78 | 0.80 | 0.79 | −0.61 | 0.92 | 0.94 |

**Figure 3** Scatter plots of (**a**) WC values and (**b**) PD values for the left *vs* right breast. Linear regression is indicated by the solid line in both plots.

**Figure 4** Scatter plot of <T2> *vs* mean WC. Linear regression is indicated by the solid line.

anthropometric variables investigated, significant correlations ($P<0.05$) were only found between <T2> and body weight ($r_s=0.378$), and mean WC and body weight ($r_s=-0.33$), and are not included in Table II.

### Discussion

This study shows strong correlations between two objective MR parameters of breast tissue, WC and <T2>, and the PD parameter determined using semiautomated analysis of digitised X-ray mammograms. The WC and PD values of the right and left breast exhibit a strong bilateral symmetry, an established feature of asymptomatic breast parenchyma (Byng *et al.*, 1995). Both MR parameters and PD also show correlations with sociodemographic and anthropometric variables that are risk factors for breast cancer. Previous studies have shown that PD can be regarded as a strong risk factor for breast cancer (Byng *et al.*, 1994); consequently, both MR parameters exhibit potential for assessment of breast cancer risk.

**Table II** Relationships between MR parameters and selected sociodemographic variables[a,b]

| *Variable* | *Category* | *Number* | *Mean WC (%)* | *<T2> (ms)* | *Mean PD (%)* |
|---|---|---|---|---|---|
| Cancer in relatives | Yes | 26 | – | 128 | – |
| | No | 12 | – | 143 | – |
| Use of female hormones | Yes | 4 | 51 | – | 72 |
| | No | 37 | 32 | – | 50 |
| Past history of smoking | Yes | 17 | 47 | – | 62 |
| | No | 24 | 32 | – | 47 |
| Husband's occupation | Professional | 11 | 45 | – | 64 |
| | Non-professional | 10 | 32 | – | 47 |
| Education[c] | High school | 11 | 30 | – | 40 |
| | College | 13 | 32 | – | 53 |
| | University | 18 | 43 | – | 62 |

[a]Mann–Whitney test ($P<0.05$). [b]Median parameter values are shown. Parameter values are replaced by a dash (–) where no association was found. [c]Kruskal–Wallis test ($P<0.05$)

The relationship between WC and PD is quite logical. Comparison of PD with histology has indicated that PD is associated with greater proportions of collagen and epithelium, and lesser proportions of fat (Boyd *et al.*, 1992). A positive correlation between WC and PD is expected, from the situation in which the breast is composed primarily of adipose tissue (low WC value), to the situation in which the breast is composed primarily of fibroglandular tissue (high WC value). In addition, the extreme values of WC in Figure 3a of 20% and 75% are in good agreement with independent measurements of the water content in adipose and fibroglandular breast tissues within the body (Woodward and White, 1986).

The relationship between <T2> and PD is the inverse of that for WC and PD, because the T2 of adipose tissue is larger than the T2 of fibroglandular tissue. As PD increases from 0 to 100%, <T2> decreases from the value for adipose tissue to that for fibroglandular tissue. The values of <T2> reported here are qualitatively consistent with previous measurements of human breast tissue and adipose tissue, although to date, the detailed T2 properties of these tissues remain poorly characterised (Bottomley *et al.*, 1984). The <T2> value is derived from analysis of the T2 distribution of breast tissue, which contains multiple components (e.g. Figure 1d). One study at a magnetic field strength of 1.4 T reported <T2> values ranging from 87 to 157 ms although no correlation between fibroglandular tissue content and <T2> was observed (Small *et al.*, 1983). A related study at 1.4 T investigating the multiple T2 relaxation components of breast tissue found a progressive increase in T2 component values with increasing fat content, although the relative weightings of the T2 components were not reported (McSweeney *et al.*, 1984). In these studies, however, T2 decay data were fitted with a weighted sum of two or three exponentials, which are known to provide inaccurate estimates of T2 distributions of tissues (Whittall and MacKay, 1989). Analysis of tissue T2 decay as a continuous distribution of relaxation times is thought to be more appropriate, because it accounts for the larger number of T2 values arising from tissue heterogeneity. Further, detailed investigations to verify the multicomponent T2 distribution of breast tissues are warranted.

Ideally, WC and <T2> would be complementary parameters, each accounting for some separate fraction of breast cancer incidence. Otherwise, measurement of both parameters would be redundant. The two parameters do differ on a physical basis: WC measures volumetric water content, whereas <T2> reflects molecular dynamics of water and fat molecules. While WC and <T2> are both significantly associated with the anthropometric variable weight, the possibility that WC and <T2> are complementary is suggested by the differences in the correlations between WC or <T2> and PD in Table I, and the different association of the MR parameters with sociodemographic variables that are risk factors for breast cancer (Table II).

No firm conclusions concerning larger populations of women can be drawn from the results shown in Table II as a result of the small number of subjects and potential bias for some factors in subject selection. Notwithstanding this proviso, it is observed that WC and PD are associated with the same sociodemographic variables, suggesting that WC and PD may be sensitive to the same biological mechanisms that influence breast cancer risk. In contrast, <T2> is associated with a single sociodemographic variable (cancer in relatives), with which both WC and PD show no association. That further similar associations are not seen for <T2> and WC is likely due to the poorer reproducibility of the T2 measurements *vs* water content measurements (10% *vs* 1%). It is also possible that the association found for <T2> could arise by chance. The speculation that <T2> may provide complementary risk assessment is, however, not unreasonable. It is interesting that in all instances the associations found with WC, <T2>, and PD in Table II are in the same direction as their putative relationship to risk of breast cancer. Thus, women with a past history of smoking, a factor associated with increased risk of breast cancer in premenopausal women (Schechter *et al.*, 1985), exhibited increased WC and PD values over non-smokers. Women with cancer in relatives, another factor associated with increased risk, exhibited decreased <T2> over those with no cancer in relatives, in accordance with the inverse correlation between <T2> and WC and PD. The anthropometric variable weight also showed a negative correlation with WC and a positive association with <T2>; leanness has been associated with increased risk of breast cancer in premenopausal women (Hunter and Willett, 1993).

There is further indirect evidence that the T2 distribution of bulk breast tissue may contain additional information over PD and WC values. The majority of the subjects in this study (29 of 38) exhibited a T2 distribution consisting of three relaxation components. The mean and standard deviation of the T2 values of each component were $34 \pm 12$, $105 \pm 29$ and $337 \pm 114$ ms, with fractional weightings of $25 \pm 11$, $53 \pm 9$ and $22 \pm 12\%$ respectively. The T2 distribution results from spatial averaging of T2 values for the different tissues in the breast parenchyma, and the multiple T2 components associated with the individual tissues themselves. Recent studies have suggested that analysis of multicomponent T2 relaxation in tissues may provide clinical applications. For example, a T2 component of white matter may provide quantitative indication of demyelinating diseases such as multiple sclerosis (MacKay *et al.*, 1994). Accurate determination of the T2 relaxation distribution of breast tissue may provide analogous tissue characterisation, and information different from that provided by WC, e.g. the capability to distinguish between the epithelial and stromal components of fibroglandular tissue.

In this study, however, no statistically significant correlation was found between T2 components and PD. This result is primarily related to inadequacies in the quality of the measured T2 decay data. The estimation of multicomponent T2 distributions from T2 decay data is a difficult problem that depends crucially on the signal-to-noise ratio, the number of data samples, and the echo time. Analysis of simulated data has indicated that the T2 distribution can be resolved coarsely under the experimental conditions present in the study, but that the statistical uncertainty associated with the fitting procedure remains too large for accurate determination of subtle changes in individual T2 components (Graham *et al.*, 1995*b*). There is no doubt that higher signal-to-noise ratio, acquisition of more than 70 data points, and shorter echo time would be very beneficial in determining the T2 distribution more accurately. This provides motivation for further improvements in measurement technique. In addition, a detailed investigation of the T2 distribution of breast tissue *ex vivo* is currently being conducted, using a dedicated MR spectrometer to provide T2 decay data of high quality. This investigation should identify which features of the T2 distribution correspond to fibroglandular and adipose tissue, and how the distribution varies for different fibroglandular fractions.

The strength of the MR parameters as risk factors for breast cancer cannot be determined from this group of asymptomatic subjects and requires the implementation of a case–control study. Additional, important aspects of such a study would be to determine the strength of the MR parameters as risk factors in relation to their X-ray counterparts, and to determine the fraction of breast cancer incidence that can be accounted for using MR and X-ray parameters in combination. The possibility that MR parameters provide an enhanced measure of risk could have important implications for epidemiological studies of breast cancer. Even if this potential outcome is not validated, the MR measurements still have utility owing to the non-hazardous nature of the examination, and the objective nature of the derived parameters.

In particular, the non-hazardous nature of the MR examination permits investigation of the breast parenchyma at an increased frequency of measurement and with reduced risk to trial subjects compared with X-ray mammography. For example, MR measurements could be used to investigate the development of the breast parenchymal patterns with age in high risk subjects, or to assess potential preventative intervention strategies, such as the use of hormone supplements for reducing risk of breast cancer (Spicer *et al.*, 1994), use of tamoxifen (Nayfield *et al.*, 1991) or dietary modification (Boyd *et al.*, 1990). The role of MR parameters in determining strategies for breast cancer screening remain to be determined. The suggestion that PD could be used to influence the frequency of screening intervals is controversial (Tabar and Dean, 1982) and has not been investigated to date. A similar use of MR requires further experimental evidence, and also a cost benefit analysis.

**Acknowledgements**

The authors thank Dr Ralph Blend for his assistance in use of the MR imaging facility at Princess Margaret Hospital, Toronto; Doris Moro, Peter Saranchuk and Suzanne Bradshaw for imaging the subjects; Laura Sagar for subject recruitment; Laurie Little for digitisation of mammograms and statistical analysis; John Watts for construction of breast coils and John Snider, Ian Gage and John Bock of General Electric Medical Systems, Canada, for technical assistance. This work was supported by a Terry Fox Programme Project grant from the National Cancer Institute of Canada and General Electric Medical Systems, Canada. SJ Graham was supported by a scholarship from the Natural Sciences and Engineering Research Council of Canada.

### References

BOTTOMLEY PA, FOSTER TH, ARGERSINGER RE AND PFEIFER LM. (1984). A review of normal tissue hydrogen NMR relaxation times and relaxation mechanisms from 1–100 MHz: dependence on tissue type, NMR frequency, temperature, species, excision, and age. *Med. Phys.*, **11,** 425–447.

BOYD NF, COUSINS M, LOCKWOOD G AND TRITCHLER D. (1990). The feasibility of testing experimentally the dietary fat breast cancer hypothesis. *Br. J. Cancer*, **62,** 878–881.

BOYD NF, JENSEN HM, COOKE G AND LEE HAN H. (1992). Relationship between mammographic and histological risk factors for breast cancer. *J. Natl Cancer Inst.*, **84**, 1170–1179.

BOYD NF, BYNG JW, JONG RA, FISHELL EK, LITTLE LE, MILLER AB, LOCKWOOD GA, TRITCHLER DL AND YAFFE MJ. (1995). Quantitative classification of mammographic densities and breast cancer risk: results from the Canadian National Breast Screening Study. *J. Natl Cancer Inst.*, **87**, 670–675.

BRISSON JB, MERLETTI F, SADOWSKI NL, TWADDLE JA, MORRISON AS AND COLE P. (1982). Mammographic features of the breast and breast cancer risk. *Am. J. Epidemiol.*, **115**, 428–437.

BYNG JW, BOYD NF, FISHELL E, JONG RA AND YAFFE MJ. (1994). The quantitative analysis of mammographic densities. *Phys. Med. Biol.*, **39**, 1629–1638.

BYNG JW, BOYD NF, LITTLE L, LOCKWOOD G, FISHELL E, JONG RA AND YAFFE MJ. (1995). Symmetry of projection in the quantitative analysis of mammographic images. *Eur. J. Cancer Prev.*, (submitted).

CARR HY AND PURCELL EM. (1954). Effects of diffusion on free precession in nuclear magnetic resonance experiments. *Phys. Rev.*, **94**, 630–638.

DIXON WT. (1984). Simple proton spectroscopic imaging. *Radiology*, **153**, 189–194.

GOODWIN PJ AND BOYD NF. (1988). Mammographic parenchymal pattern and breast cancer risk: a critical appraisal of the evidence. *Am. J. Epidemiol.*, **127**, 1097–1108.

GRAHAM SJ AND BRONSKILL MJ. (1995). MR measurement of relative water content and multicomponent T2 relaxation times applied to human breast. *Magn. Reson. Med.*, (in press).

GRAHAM SJ, STANCHEV PL, LLOYD-SMITH JOA AND BRONSKILL MJ. (1995*a*). Changes in fibroglandular volume and water content of breast tissue during the menstrual cycle observed by magnetic resonance imaging at 1.5 T. *J. Magn. Reson. Imaging*, **5**, 695–701.

GRAHAM SJ, STANCHEV PL AND BRONSKILL MJ. (1995*b*). Multicomponent T2 Relaxation analysis of data measured on clinical MR scanners. *Magn. Reson. Med.*, (in press).

HAYS WL AND WINKLER RL. (1971). *Statistics: Probability, Inference, and Decision*. Holt, Rinehart & Winston: New York.

HORE PJ. (1983). Solvent suppression in Fourier transform nuclear magnetic resonance. *J. Magn. Reson.*, **55**, 283–300.

HUNTER DJ AND WILLETT WC. (1993). Diet, body size and breast cancer. *Epidemiol. Rev.*, **15**, 110–132.

KAIZER L, FISHELL EK, HUNT JW, FOSTER FS AND BOYD NF. (1988). Ultrasonographically defined parenchymal patterns of the breast: relationship to mammographic patterns and other risk factors for breast cancer. *Br. J. Radiol.*, **61**, 118–124.

KELSEY JL, HILDRETH NG AND THOMPSON WD. (1983). Epidemiologic aspects of breast cancer. *Radiol. Clin. North Am.*, **21**, 3–12.

MACKAY AL, WHITTALL KP, ADLER J, LI D, PATY DW AND GRAEB D. (1994). *In vivo* visualization of myelin water in brain by magnetic resonance. *Magn. Reson. Med.*, **31**, 673–677.

MCSWEENEY MB, SMALL WC, CERNY V, SEWELL W, POWELL RW AND GOLDSTEIN JH. (1984). Magnetic resonance imaging in the diagnosis of breast disease: use of transverse relaxation times. *Radiology*, **153**, 741–744.

MEIBOOM S AND GILL D. (1958). Modified spin-echo method for measuring nuclear relaxation times. *Rev. Sci. Instr.*, **29**, 688–691.

MIKI Y, SWENSEN J, SHATTUCK-EIDENS D, FUTREAL PA, HARSHMAN K, TAVTIGIAN S, LIU Q, COCHRAN C, BENNETT LM, DING W, BELL R, ROSENTHAL J, HUSSEY C, TRAN T, MCCLURE M, FRYE C, HATTIER T, PHELPS R, HAUGEN-STRANO A, KATCHER H, YAKUMO K, GHOLAMI Z, SHAFFER D, STONE S, BAYER S, WRAY C, BOGDEN R, DAYANANTH P, WARD J, TONIN P, NAROD S, BRISTOW PK, NORRIS FH, HELVERING L, MORRISON P, ROSTECK P, LAI M, BARRETT JC, LEWIS C, NEUHAUSEN S, CANNON-ALBRIGHT L, GOLDGAR D, WISEMAN R, KAMB A AND SKOLNICK MH.(1994). A strong candidate for the breast and ovarian cancer susceptibility gene BRCA1. *Science*, **266**, 66–71.

MILLER AB, BAINES CJ, TO T AND WALL C. (1992*a*). Canadian National Breast Screening Study.2. breast cancer detection and death rates among women aged 50 to 59 years. *Can. Med. Assoc. J.*, **147**, 1477–1488.

MILLER AB, BAINES CJ, TO T AND WALL C. (1992*b*). Canadian National Breast Screening Study.1. breast cancer detection and death rates among women aged 40 to 49 years. *Can. Med. Assoc. J.*, **147**, 1459–1476.

NAYFIELD SG, KARP JE, FORD LG, DORR FA AND KRAMER BS. (1991). Potential role of tamoxifen in prevention of breast cancer. *J. Natl Cancer Inst.*, **83**, 1450–1459.

POON CS, SZUMOWSKI J, PLEWES DB, ASHBY P AND HENKELMAN RM. (1989). Fat/water quantitation and differential relaxation time measurement using chemical shift imaging technique. *Magn. Reson. Imaging*, **7**, 369–382.

POON CS, BRONSKILL MJ, HENKELMAN RM AND BOYD NF. (1992). Quantitative magnetic resonance parameters and their relationship to mammographic pattern. *J. Natl Cancer Inst.*, **84**, 777–781.

SAFTLAS AF AND SZKLO M. (1987). Mammographic parenchymal patterns and breast cancer risk. *Epidemiol. Rev.*, **9**, 146–174.

SCHECHTER MT, MILLER AB AND HOWE GR. (1985). Cigarette smoking and breast cancer: a case–control study of screening program participants. *Am. J. Epidemiol.*, **121**, 479–487.

SEIDMAN H, STELLMAN S AND MUSHINKI MH. (1982). A different perspective on breast cancer risk factors: some implications of the nonattributable risk. *CA*, **32**, 301–313.

SMALL WC, MCSWEENEY MB, GOLDSTEIN JH, SEWELL W AND POWELL RW. (1983). Handling of *in vitro* human breast tissue samples: protocol requirements for accurate NMR relaxation measurements. *Biochem. Biophys. Res. Commun.*, **112**, 991–999.

SPICER DV, URSIN G, PARISKY YR, PEARCE JG, SHOUPE D, PIKE A AND PIKE MC. (1994). Changes in mammographic densities induced by a hormonal contraceptive designed to reduce breast cancer risk. *J. Natl Cancer Inst.*, **86**, 431–436.

TABAR L AND DEAN PB. (1982). Mammographic parenchymal patterns: risk indicator for breast cancer? *JAMA*, **247**, 185–189.

WARNER E, LOCKWOOD G, TRITCHLER D AND BOYD NF. (1992). The risk of breast cancer associated with mammographic parenchymal patterns: a meta-analysis of the published literature to examine the effect of method of classification. *Cancer Detect. Prev.*, **16**, 67–72.

WHITTALL KP AND MACKAY AL. (1989). Quantitative interpretation of NMR relaxation data. *J. Magn. Reson.*, **84**, 134–152.

WOLFE JN. (1976). Breast patterns as an index of risk of developing breast cancer. *Am. J. Roentgenol.*, **126**, 1130–1139.

WOODARD HQ AND WHITE DR. (1986). The composition of body tissues. *Br. J. Radiol.*, **59**, 1209–1219.

WOOSTER R, NEUHAUSEN SL, MANGION J, QUIRK Y, FORD D, COLLINS N, NGUYEN K, SEAL S, TRAN T, AVERILL D, FIELDS P, MARSHALL G, NAROD S, LENOIR GM, LYNCH H, FEUNTEUN J, DEVILEE P, CORNELISSE CJ, MENKO FH, DALY PA, ORMISTON W, MCMANUS R, PYE C, LEWIS CM, CANNON-ALBRIGHT LA, PETO J, PONDER BAJ, SKOLNICK MH, EASTON DF, GOLDGAR DE AND STRATTON MR. (1994). Localization of a breast cancer susceptibility gene, BRCA2, to chromosome 13q12-13. *Science*, **265**, 2088–2090.

YIN F-F, GIGER ML, DOI K, YOSHIMURA H, XU X-W AND NISHIKAWA RM. (1992). Evaluation of imaging properties of a laser film digitizer. *Phys. Med. Biol.*, **37**, 273–280.